# MOBILE APP INTERVENTION TO PROMOTE BREAST CANCER SCREENING IN OLDER KOREAN AMERICAN WOMEN

**DOI:** 10.1093/geroni/igz038.1512

**Published:** 2019-11-08

**Authors:** Hee Y Lee

**Affiliations:** The University of Alabama, Tuscaloosa, Tuscaloosa, Alabama, United States

## Abstract

To promote breast cancer screening behavior in older Korean American women, a mobile phone multimedia messaging intervention (mMammogram) was developed. The current study explored how the mMammogram intervention can be applied and improved for wide dissemination and implementation in other older racial/ethnic immigrant populations. Three focus groups were conducted with 14 older Korean American women who completed the mMammogram. Three themes emerged: (1) emphasis on knowledge improvement (understanding of breast cancer screening); (2) assistance of bilingual health navigators to promote mammography (e.g., setting up appointment and transportation); and (3) consideration of technology literacy (e.g., training for use of smartphone and connectivity issues). Mobile app intervention combined with bilingual health navigation services can be an acceptable and effective intervention medium to promote mammogram, which will bring about a positive change in women’s health screening behavior, especially for newly arrived immigrant populations.

